# Quantitative contrast enhancement volume on immediate post-thrombectomy CT predicts symptomatic intracranial hemorrhage and functional outcomes in acute large vessel occlusion stroke

**DOI:** 10.3389/fneur.2025.1579659

**Published:** 2025-05-29

**Authors:** Ziyang Huang, Zhiyu Xiong, Chen Gong, Shuyu Jiang, Liping Huang, You Wang, Jinxian Yuan, Yuan Gao, Yuenan Ban, Yangmei Chen, Tao Xu

**Affiliations:** ^1^Department of Neurology, The Second Affiliated Hospital of Chongqing Medical University, Chongqing, China; ^2^Chongqing Shapingba Hospital, School of Medicine, Chongqing University, Chongqing, China; ^3^Key Laboratory of Major Brain Disease and Aging Research (Ministry of Education), Chongqing Medical University, Chongqing, China

**Keywords:** endovascular treatment, acute ischemic stroke, contrast enhancement, symptomatic intracranial hemorrhage, functional independence

## Abstract

**Objectives:**

Symptomatic intracranial hemorrhage (sICH) following endovascular thrombectomy (EVT) for acute ischemic stroke due to anterior circulation large vessel occlusion (AIS-LVO) significantly impacts clinical outcomes. Contrast enhancement (CE) on immediate post-EVT non-contrast CT (NCCT) may reflect blood–brain barrier disruption, but its volumetric correlation with sICH and functional independence remains underexplored.

**Methods:**

We performed a retrospective screening on consecutive AIS-LVO patients who had CE on NCCT immediately within 2 h after EVT. The quantitative volume of CE was calculated by using 3D Slicer software. Multivariable logistic regression was performed to achieve the risk factors of sICH and functional independence. The discrimination and calibration of the multivariable models were assessed using the area under the receiver operator characteristic curve, fivefold cross-validation, calibration curve, and decision curve analysis.

**Results:**

In this study, 111 patients were enrolled in the final analysis. According to the restricted cubic spline, 10.6 mL was the optimal threshold of CE volume dichotomization for patients with AIS-LVO. In multivariate regression analysis, the CE_+_ group (CE volume beyond 10.6 mL) was significantly associated with sICH (aOR: 5.24, 95% CI: 1.45–18.99, *p* = 0.012) and functional independence (10.9% vs. 51.8%; aOR 0.05, 95% CI: 0.01–0.28, *p* < 0.001). The multivariable models demonstrated good discrimination and calibration in this cohort, as well as the fivefold cross-validation.

**Conclusion:**

Volumetric quantification of CE on immediate post-EVT NCCT serves as a novel biomarker for early sICH risk stratification and functional prognosis in AIS-LVO. Incorporating CE volume into predictive models enhances clinical utility, enabling timely diagnosis and intervention.

## Introduction

The global burden of acute ischemic stroke (AIS) continues to escalate in aging populations, remaining a predominant cause of disability and mortality (1). Endovascular treatment (EVT) has emerged as the standard care for anterior circulation large vessel occlusion (AIS-LVO) based on current guidelines (2). Nevertheless, functional independence is not attained in nearly half of AIS-LVO patients who underwent EVT (3), a phenomenon potentially attributable to procedure-related complications. Intracranial hemorrhage (ICH) after EVT is a common and terrible complication due to the injury of cerebral vascular endothelium and blood–brain barrier (4), which may be associated with poor clinical outcomes among AIS-LVO patients (5).

While recent investigations have elucidated risk factors for post-EVT hemorrhagic transformation (6–9), reliable early predictors remain elusive. Immediate post-procedural non-contrast computed tomography (NCCT) frequently reveals contrast enhancement (CE) phenomena in AIS-LVO patients (10), with a reported incidence ranging from 30.7 to 87.5% (11). Furthermore, both contrast extravasation and intracranial hemorrhage manifest as hyperdense areas on NCCT imaging (12), and a followed NCCT scan performed at least 24 h after EVT is required to distinguish them (13). This diagnostic latency underscores the need for novel predictive biomarkers.

Emerging evidence suggests that a larger area of CE, described by the Alberta Stroke Program Early Computed Tomography Score (ASPECTS) (14), was associated with symptomatic ICH (sICH), which may demonstrate more severe disruption of blood–brain barrier (11). Notably, the degree of blood–brain barrier injury is associated with the severity of ICH (15). Therefore, the quantitative volume of CE warrants further exploration to ascertain its potential association with sICH and functional independence.

In this context, to diagnose ICH as early as possible, the present study used the NCCT immediately after EVT to delineate and calculate the quantitative volume among AIS-LVO patients. We hypothesized that the volume of CE could be correlated with sICH and functional independence in patients undergoing EVT.

## Methods

### Patient selection

From February 2019 to February 2022, we retrospectively identified consecutive AIS-LVO patients who underwent EVT in the Second Affiliated Hospital of Chongqing Medical University. Electronic medical records and imaging information were obtained by the hospital information system. Inclusion criteria were: (1) age≥18 years; (2) diagnosis as AIS-LVO confirmed by head digital subtraction angiography and receiving EVT within 24 h from stroke onset; and (3) presence of contrast enhancement (CE) on NCCT within 2 hours after EVT, which was difficultly distinguished between the CE and hemorrhage by two experienced interventional neuroradiologist. Patients were excluded with the following criteria: (1) who did not receive NCCT within 2 hours after thrombectomy; (2) failed to follow-up; or (3) inadequate clinical data. The Second Affiliated Hospital of Chongqing Medical University Human Research Ethics Committee has approved the study protocol.

### Data collection

Demographic information and baseline clinical characteristics of all eligible patients were extracted, including age, sex, medical history [smoking, drinking, hypertension, diabetes mellitus, atrial fibrillation (AF)], systolic blood pressure (SBP) at admission, diastolic blood pressure (DBP) at admission, blood glucose at admission, activated partial thromboplastin time (APTT), prothrombin time (PT), international normalized ratio (INR), intravenous thrombolysis, baseline National Institutes of Health Stroke Scale (NIHSS) scores at admission, the Trial of ORG 10172 in Acute Stroke Treatment (TOAST) classification (16), pre-stroke modified Rankin Scale (mRS) score, occlusion site, passes of stent retriever and aspiration. The time from stroke onset to groin puncture (OTP), and the time from stroke onset to revascularization (OTR) were also recorded. Baseline ischemic injury in the anterior circulation was assessed according to the Alberta Stroke Program Early Computed Tomography Score (ASPECTS) (14). The quality of reperfusion was evaluated using the extended thrombolysis in cerebral infarction (eTICI) score on the final angiogram (17). Collateral vessel status was assessed by the American Society of Interventional and Therapeutic Neuroradiology/Society of Interventional Radiology (ASITN/SIR) collateral vessel grading system (18).

### Imaging analysis

**Figure 1:**
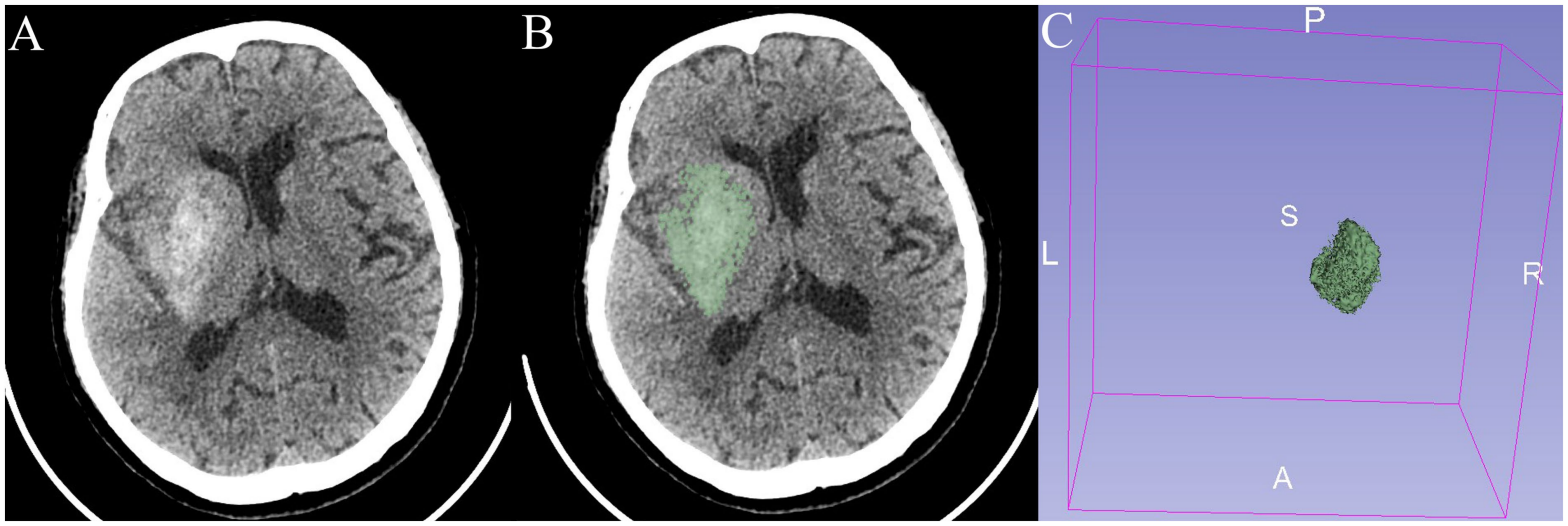
An 82-year-old patient presented with a right-sided cerebral ischemic stroke. **(A,B)** Non-contrast CT immediately after EVT shows contrast enhancement (CE). **(C)** The three-dimensional structure of CE in the brain is reconstructed with a volume of 24.9 mL.

Non-contrast brain CT (1.00/1.25 mm axial images; SOMATOM Force, German) was used in this study. CE was defined as the presence of high density on NCCT immediately after EVT. The CE volume was measured by automated 3D-Slicer software. The CT data of the patient in Digital Imaging and Communications in Medicine (DICOM) format was imported into the 3D Slicer software (3D Slicer 5.4.0). By using the threshold range that was manually set according to the HU value of CE for masking and switching to the paint effect, the pixels in which CE was present were further manually marked. Then, the three-dimensional reconstruction of the CE was realized, and the volume of the CE was calculated automatically (Figure 1) (19).

### Definition and clinical outcomes

CE was defined as the presence of high density on NCCT immediately after EVT, but with no longer discernible high density on 24 h follow-up NCCT after EVT or with no hyposignal on 24 h follow-up MRI after EVT. Hemorrhage was defined as the presence of high density on NCCT immediately after EVT, with high density on 24 h follow-up NECT after EVT or with hyposignal on 24 h follow-up MRI after EVT (10).

The primary outcomes were the incidence of sICH within 48 h after EVT and functional independence. The sICH was assessed based on follow-up CT or MRI according to the European Cooperative Acute Stroke Study (ECASS) II definition with a decline in NIHSS by 4 points (20). Functional independence was defined as mRS 0–2 at 90 days. The secondary outcomes included the incidence of ICH within 48 h, 90-day mortality, 90-day mRS 0–1 and 90-day mRS 0–3.

### Statistical analysis

Among all enrolled patients, the relationship between the CE volume and functional independence was evaluated by restricted cubic spline. After identifying the suitable cutoff of CE volume by restricted cubic spline by regression analysis, all these included patients were divided into the CE_+_ group and the CE_−_ group. The patient characteristics, imaging data, and treatment outcomes were compared between the two groups.

Significant factors (*p* < 0.10) and those linked to functional outcomes in previous studies were selected through univariable analysis. For dichotomized outcomes, multiple logistic regression was performed, adjusting for age, pre-stroke mRS, baseline NIHSS and ASPECT score, ASITN/SIR grade, mTICI score, and occlusion site.

To ensure the reliability of the models, we performed the analysis as follows. Firstly, the multicollinearity of predictors was analyzed utilizing the variance inflation factor and tolerance statistic to ascertain the reliability of the model. Secondly, the predictive power of the model for symptomatic intracranial hemorrhage and functional independence was further verified through the area under the receiver operator characteristic curve (AUC). This was done to compare the difference in predictive performance between traditional models and new models. Thirdly, internal cross-validation was carried out to assess the discriminatory performance of the scoring tool using fivefold cross-validation. Fourthly, calibration and clinical utility were evaluated by employing a calibration curve and decision curve analysis. Statistical analysis was performed using SPSS software (version 26.0; IBM SPSS Statistics) and R software (version 4.2.2). *p* < 0.05 was considered statistically significant.

## Results

### Patient characteristics

**Figure 2:**
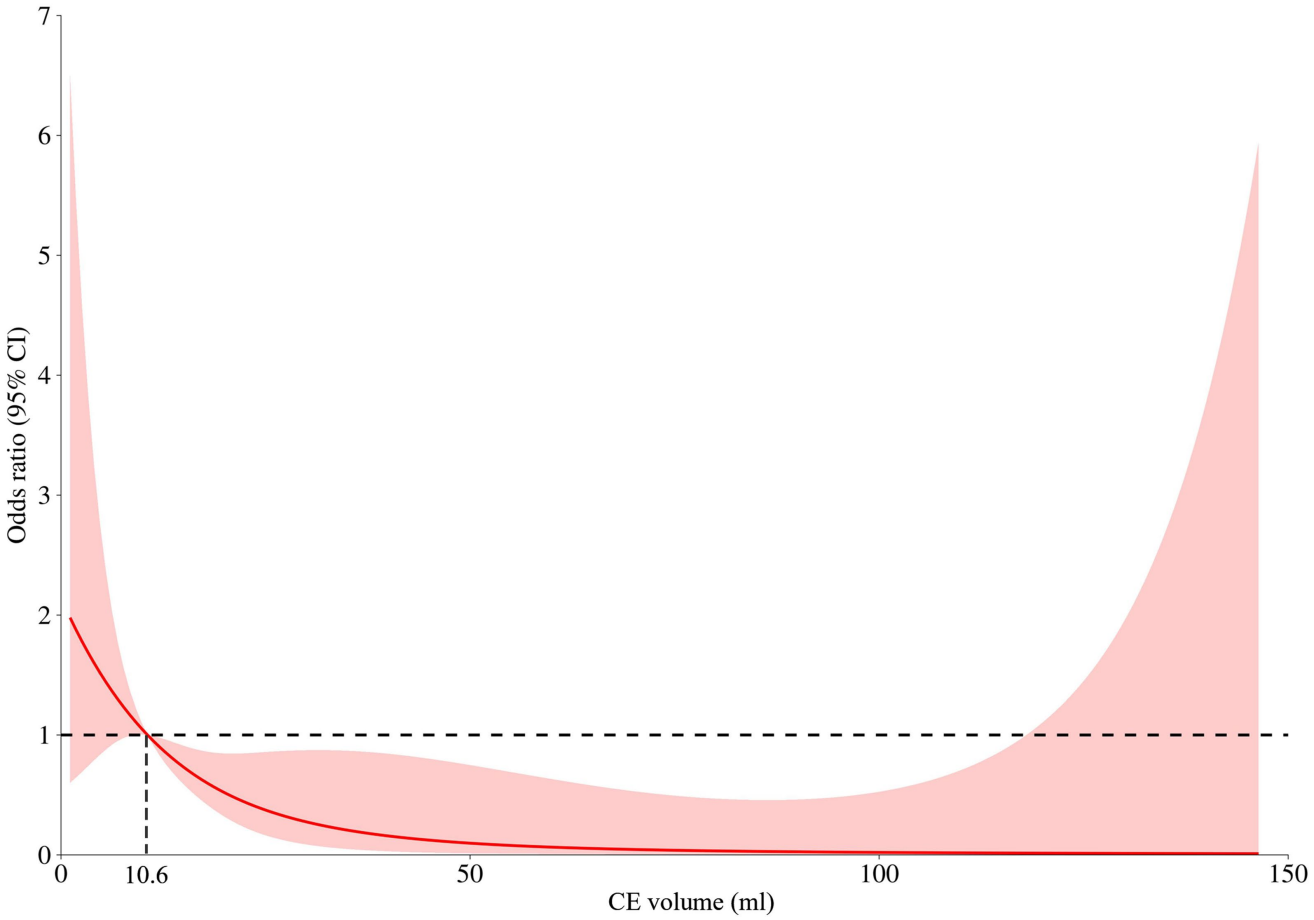
Restricted cubic spline for contrast enhancement (CE) volume and the risk of functional independence (90-day mRS 0–2). A nonlinear relationship between the contrast enhancement volume and risk of functional outcomes was observed and CE produced a significant risk for functional independence as the volume of CE exceeded 10.6 mL.

We retrospectively included 153 consecutive anterior circulation AIS-LVO patients, of which 111 (72.5%) with CE on NCCT within 2 hours after EVT enrolled in the current analysis. According to the restricted cubic spline (Figure 2), all these included patients were divided into the CE_+_ (CE volume ≥ 10.6 mL) group and the CE_−_ group (CE volume < 10.6 mL). As shown in Table 1, the median age was 67 years, and 51.4% of them were men. Compared to CE_−_ patients, CE_+_ patients presented with higher baseline NIHSS score (median [interquartile range (IQR)], 17 [11–22] vs. 13 [8–16]; *p* < 0.001), baseline ASPECTS (median [IQR], 6 [5–7] vs. 7 [6–8]; *p* < 0.001), lower ASITN/SIR score (median [IQR], 1 [0–2] vs. 2 [1–3]; *p* = 0.020) and larger CE volume (median [IQR], 42.1 [20.6–86.4] vs. 5.2 [1.9–8.2]; *p* < 0.001). Other baseline characteristics did not differ significantly between the two groups (*p* > 0.05; Table 1).

**Table 1:** Baseline characteristics according to groups of CE_+_ and CE_−_.

Characteristics	All (*n* = 111)	CE_+_ (*n* = 55)	CE_−_ (*n* = 56)	*p* value
Age, median (IQR)	67 (60–77)	69 (60–78)	68 (60–82)	0.457
Sex, *n* (%)	57 (51.4)	30 (54.5)	27 (48.2)	0.505
Medical history, *n* (%)				
Smoking	43 (38.7)	22 (40.0)	21 (37.5)	0.787
Drinking	35 (31.5)	17 (30.9)	18 (32.1)	0.889
Hypertension	55 (49.5)	28 (50.9)	27 (48.2)	0.776
Diabetes mellitus	19 (17.1)	8 (14.3)	11 (20.0)	0.424
Atrial fibrillation	49 (44.1)	26 (47.3)	23 (41.1)	0.511
Clinical characteristics				
SBP admission, mmHg; mean (SD)	140 (129–159)	150 (133–163)	135 (133–154)	0.158
DBP admission, mmHg; mean (SD)	80 (74–90)	80 (74–89)	83 (76–95)	0.103
blood glucose, median (IQR)	7.2 (6.2–8.8)	7.2 (6.2–9.1)	7.1 (6.0–8.5)	0.559
APTT, median (IQR)	34.7 (31.8–37.9)	35 (31–39)	35 (32–37)	0.935
PT, median (IQR)	13.4 (12.9–15.2)	13.5 (12.9–15.7)	13.3 (12.8–15.0)	0.527
INR, median (IQR)	0.99 (0.95–15.2)	1.00 (0.96–1.12)	0.99 (0.93–1.10)	0.209
Pre-stroke mRS, median (IQR)	0 (0–0)	0 (0–0)	0 (0–0)	0.118
TOAST, *n* (%)				0.362
Large atherosclerotic stroke	52 (46.8)	28 (50.9)	24 (42.9)	
Cardiogenic stroke	54 (48.6)	26 (47.3)	28 (50.0)	
Other	5 (4.5)	1 (18.1)	4 (7.1)	
Baseline NIHSS score, median (IQR)	15 (10–20)	17 (11–22)	13 (8–16)	<0.001
Baseline ASPECTS, median (IQR)	6 (5–7)	6 (5–7)	7 (6–8)	<0.001
ASITN/SIR score, median (IQR)	2 (0–2)	1 (0–2)	2 (1–3)	0.018
mTICI 2b-3, *n* (%)	97 (87.4)	49 (89.1)	48 (85.7)	0.592
OTP, min; median (IQR)	421 (260–749)	456 (273–714)	408 (247–810)	0.887
OTR, min; median (IQR)	525 (373–844)	571 (396–1,036)	491 (341–919)	0.445
Intravenous thrombolysis, *n* (%)	50 (45.0)	28 (50.9)	22 (39.3)	0.218
Occlusion site, *n* (%)				0.021
ICA	33 (29.7)	23 (41.8)	10 (17.9)	
MCA-1	67 (60.4)	28 (50.9)	39 (69.6)	
MCA-2	11 (9.9)	4 (7.3)	7 (12.5)	
CE volume, median (IQR)	10.6 (5.2–42.1)	42.1 (20.6–86.4)	5.2 (1.9–8.2)	<0.001

CE_+_, defined as CE volume ≥ 10.6 mL; CE_−_, defined as CE volume < 10.6 mL; SBP, systolic blood pressure; DBP, diastolic blood pressure; APTT, activated partial thromboplastin time; PT, prothrombin time; INR, international normalized ratio; mRS, modified Rankin Scale; TOAST, Trial of ORG 10172 in acute stroke treatment; NIHSS, National Institutes of Health Stroke Scale; ASPECTS, Alberta Stroke Program Early CT Score; ASITN/SIR, American Society of Interventional and Therapeutic Neuroradiology/Society of Interventional Radiology; eTICI, extended thrombolysis in cerebral infarction; OTP, the time from stroke onset to groin puncture; OTR, the time from stroke onset to revascularization; ICA, internal carotid artery; MCA, middle cerebral artery; SD, standard deviation; IQR, interquartile range; CE, contrast enhancement.

### Clinical outcomes

Significant factors (*p* < 0.10) and those linked to functional outcomes in previous studies were selected through univariable analysis, including age, pre-stroke mRS, baseline NIHSS score, baseline ASPECTS, ASITN/SIR score, eTICI 2b-3, and occlusion site were included in multivariable logistic regression analysis (Table 1; Supplementary Table S1). For primary clinical outcomes after adjusting for confounding factors, sICH occurred in 18 of 55 (32.7%) patients in the CE_+_ group and 4 of 56 (7.1%) patients in the CE_−_ group (aOR: 5.24, 95% CI: 1.45–18.99, *p* = 0.012; Table 2; Supplementary Table S2). Overall, the CE_+_ group exhibited a decreased frequency of functional independence than the CE_−_ group (10.9% vs. 51.8%; aOR 0.05, 95% CI: 0.01–0.28, *p* < 0.001; Table 2; Supplementary Table S3).

**Table 2:** Clinical outcomes according to groups of CE_+_ and CE_−_.

Characteristics	All patients			*p* value	Adjusted OR (95% CI)	*p* value
	All (*n* = 111)	CE_+_ (*n* = 55)	CE_−_ (*n* = 56)			
Primary outcomes						
sICH within 48 h, *n* (%)	22 (19.8)	18 (32.7)	4 (7.1)	<0.001	5.24 (1.45–18.99)	0.012
90-day mRS 0–2, *n* (%)	35 (31.5)	6 (10.9)	29 (51.8)	<0.001	0.05 (0.01–0.28)	<0.001
Secondary outcomes						
Any ICH within 48 h, *n* (%)	34 (30.6)	25 (45.5)	9 (16.1)	<0.001	6.45 (2.06–20.18)	0.001
90-day mortality, *n* (%)	42 (38.2)	30 (55.6)	12 (21.4)	<0.001	4.22 (1.35–13.22)	0.013
90-day mRS 0–1, *n* (%)	22 (19.8)	2 (3.6)	22 (19.8)	<0.001	0.06 (0.01–0.34)	0.002
90-day mRS 0–3, *n* (%)	43 (38.7)	12 (21.8)	31 (55.4)	<0.001	0.23 (0.07–0.74)	0.014

CE_+_, defined as CE volume ≥ 10.6 mL; CE_−_, defined as CE volume < 10.6 mL; mRS, modified Rankin Scale; ICH, intracranial hemorrhage; sICH, symptomatic intracranial hemorrhage; aOR, adjusted odd ratio; CI, confidence interval.

For secondary outcomes, there were significant differences in 90-day mRS 0–1 (aOR 0.06, 95% CI: 0.01–0.34, *p* = 0.002), 90-day mRS 0–3 (aOR 0.23, 95% CI: 0.07–0.74, *p* = 0.014), any ICH within 48 h (aOR 6.45, 95% CI: 2.06–20.18, *p* = 0.001) and 90-day mortality (aOR 4.22, 95% CI: 1.35–13.22, *p* = 0.013) between the CE_+_ group and the CE_−_ group after adjustment for confounding factors (Table 2).

### Validation of the multivariable models for sICH and functional independence

**Figure 3:**
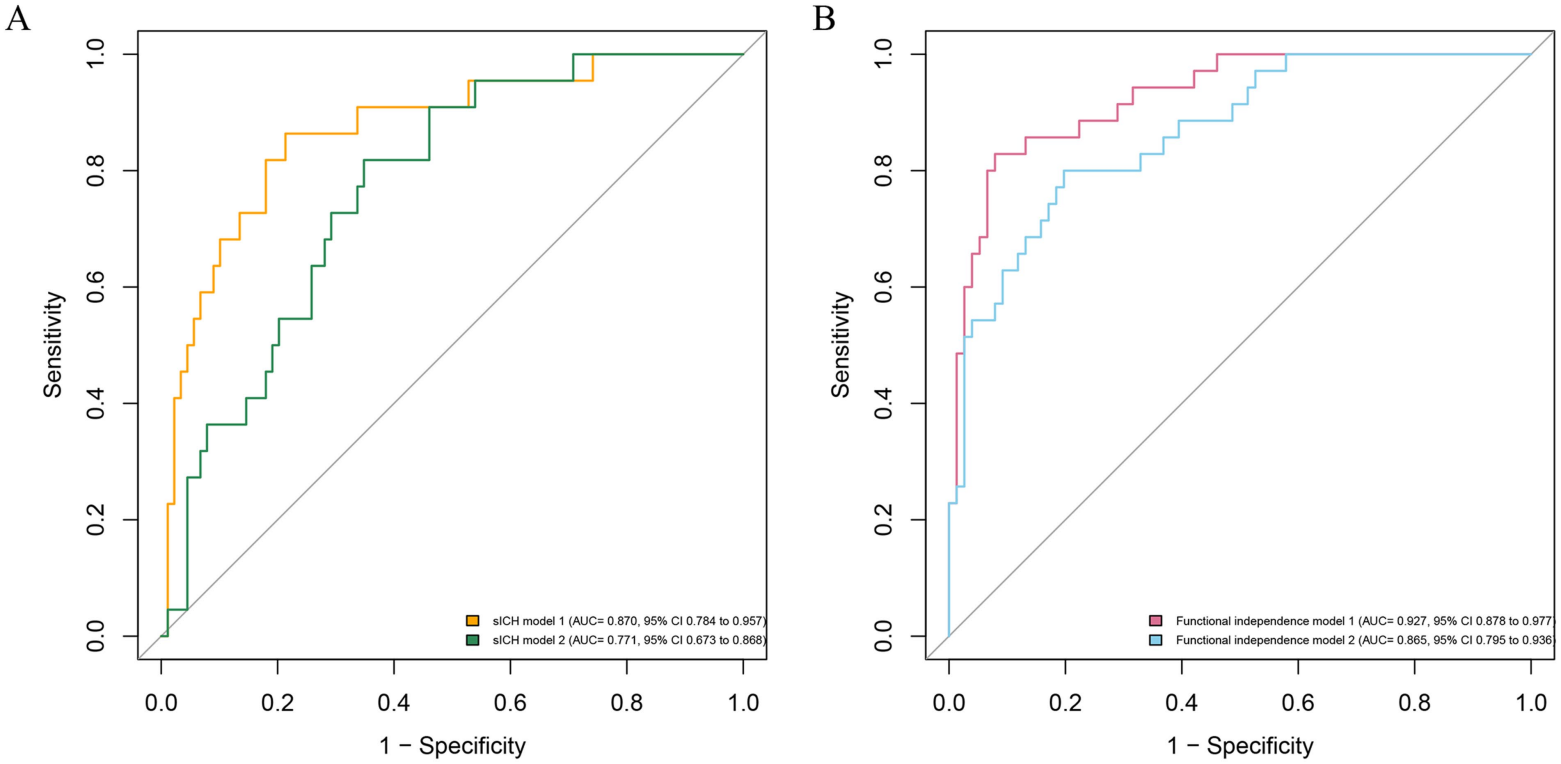
Receiver operating characteristic curve for the prediction of symptomatic intracranial hemorrhage **(A)** and functional independence **(B)**. *sICH model 2*, consisting of conventional variables, is displayed with AUC of 0.771 (95% CI 0.673 to 0.868; P<0.05); *sICH model 1*, including conventional variables plus CE volume, shows an AUC of 0.870 (95% CI 0.784 to 0.957; *p*<0.05), significantly higher than *sICH model 2* (the difference value of AUC 0.100, *p* = 0.01); *Functional independence model 2*, consisting of conventional variables, is displayed with AUC of 0.865 (95% CI 0.795 to 0.936; *p*<0.05); *Functional independence model 1*, including conventional variables plus CE volume, shows an AUC of 0.927 (95% CI 0.878 to 0.977; *p*<0.05), significantly higher than *Functional independence model 2* (the difference value of AUC 0.062, *p* = 0.02). The conventional variables include age, pre-stroke mRS, baseline NIHSS score, baseline ASPECTS, ASITN/SIR score, eTICI 2b-3, and occlusion site. sICH, symptomatic intracranial hemorrhage; AUC, area under the curve; CI, confidence interval.

**Figure 4:**
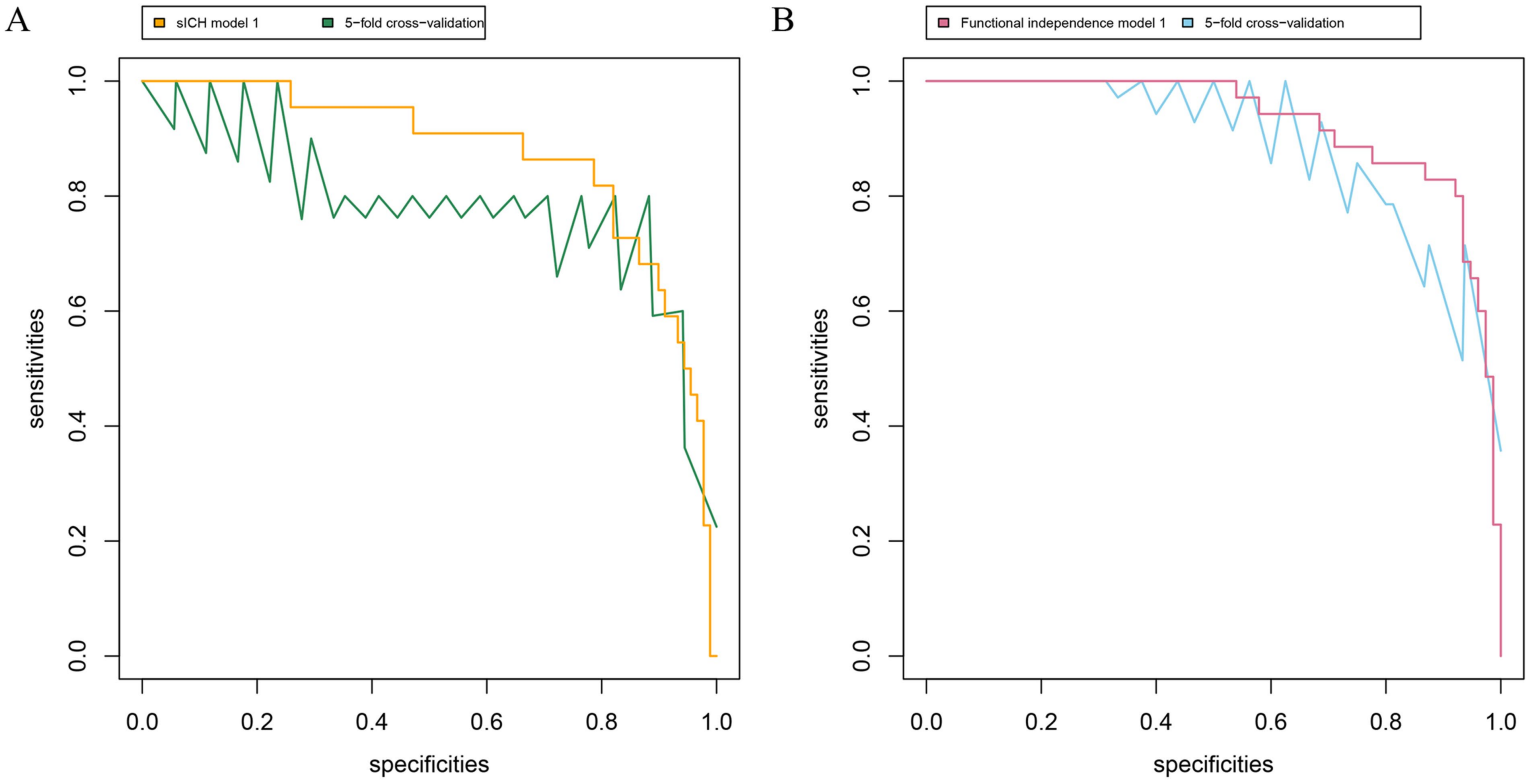
Receiver operating characteristic curve for the prediction and symptomatic intracranial hemorrhage **(A)** and functional independence **(B)**. *sICH model 1*, including conventional variables plus CE volume, shows an AUC of 0.870 (95% CI 0.784 to 0.957; *p*<0.05); *sICH model 1* based on fivefold cross validation with AUC of 0.758 (95% CI 0.526 to 0.896; *p* < 0.05). *Functional independence model 1*, including conventional variables plus CE volume, shows an AUC of 0.927 (95% CI 0.878 to 0.977; *p*<0.05); *Functional independence model 1* based on fivefold cross validation with AUC of 0.862 (95% CI 0.830 to 0.933; *p* < 0.05). The conventional variables include age, pre-stroke mRS, baseline NIHSS score, baseline ASPECTS, ASITN/SIR score, eTICI 2b-3, and occlusion site. sICH, symptomatic intracranial hemorrhage; AUC, area under the curve; CI, confidence interval.

First, the variance inflation factor (VIF) values were < 5, and tolerance values were > 0.5, suggesting no obvious collinearity in all independent variables (Supplementary Table S4). Second, sICH model 1, including conventional variables plus CE volume, showed an AUC of 0.870, significantly higher than sICH model 2 (the difference value of AUC 0.100, *p* = 0.01; Figure 3), and Functional independence model 1, including conventional variables plus CE volume, shows an AUC of 0.927, significantly higher than Functional independence model 2 (the difference value of AUC 0.062, *p* = 0.02). Third, internal cross-validation was conducted to evaluate the discriminatory performance of the scoring tool using fivefold cross validation in Figure 4 (sICH: AUC = 0.758, 95% CI 0.526 to 0.896, *p* < 0.05; functional independence: AUC = 0.862, 95% CI 0.830 to 0.933, *p* < 0.05). The calibration curve showed that the predicted and actual survival curves were close, with no significant differences (Supplementary Figure S1), indicating good overall consistency of CE volume models. Fourth, decision curve analysis showed that the sICH model 1 and Functional independence model 1 was above the horizontal, oblique lines and the traditional model curves, indicating that the predictive score provides sound clinical guidance with a better net benefit (Supplementary Figure S2).

In consideration of antithrombotic drugs usage and mean arterial pressure within 48 h after EVT, we further investigated the differences between the two groups. The results demonstrated significant differences in both mean systolic blood pressure (SBP) (median [interquartile range (IQR)], 129 [118–137] vs. 123 [111–133]; *p* < 0.05) and anticoagulant drugs usage (25.5% vs. 44.6%; *p* < 0.05) between CE + and CE- groups during the first 48 h after EVT. Furthermore, additional multivariable logistic regression analyses were performed for sICH and functional independence. The results remained consistent with our previous findings, with large volume of CE persisting as an independent risk factor for both sICH (aOR 4.56, 95% CI: 1.17–17.74, *p* = 0.029) and functional independence (aOR 0.07, 95% CI: 0.01–0.34, *p* = 0.001).

## Discussion

To our knowledge, this is the first study to explore the association between the quantitative volume of contrast enhancement (CE) and symptomatic intracranial hemorrhage (sICH) among acute ischemic stroke caused by anterior circulation large vessel occlusion (AIS-LVO) patients undergoing endovascular treatment (EVT). Our findings revealed that (1) 10.6 mL was the optimal threshold of CE dichotomization volume for patients with AIS-LVO. Patients with CE_+_ (defined as CE volume beyond 10.6 mL) experienced an increased frequency of sICH and a decreased frequency of functional independence. We verified the results by the internal fivefold cross-validation; (2) the predictive power of the new models with CE volume for sICH functional independence was significantly better than that of the traditional model.

CE is usually assessed by a non-contrast computed tomography (NCCT) scan or dual-energy CT (DECT) immediately after EVT and progressively resolves within 24 h after EVT (21, 22). In this study, the incidence of CE on NCCT after EVT was 72.5%, which was similar to previous reports (30.7–87.5%) (19), and the occurrence rates of ICH and sICH were separately 30.6 and 19.8%. Our previous meta-analysis showed that CE was associated with higher risks of ICH and poor functional outcomes in patients with AIS-LVO undergoing EVT (10). More attention needs to be focused on this postoperative manifestation. Our previous work has also indicated that a larger spatial area distribution of CE was an independent and strong risk factor for sICH (23). In this study, we calculated the quantitative volume of CE in a novel way and further found a significant association between the CE volume and a higher risk of sICH in patients with AIS-LVO after adjusting for confounding factors. In line with a recent study, the CE reliably predicted sICH in patients with CE on NCCT after EVT treatment (11). Furthermore, we created a new model including CE volume to predict sICH (AUC = 0.870), which was significantly more effective than the traditional model (24). Our study is of some value for early prediction of ICH. Through quantitative analysis, this study illustrated the predictive effect of CE on early intracranial hemorrhage and functional independence after reperfusion therapy, without having to wait 19–24 h to review the CT (13). CE volume may be an early predicting window for clinical outcomes among AIS-LVO patients.

This negative effect of CE volume on hemorrhagic transformation and functional outcomes could be due to the following reasons. First, extravasation of contrast is often attributed to disruption of the blood–brain barrier (25), whose permeability is associated with hemorrhagic transformation in the hyperacute phase and with improved functional outcomes in the late subacute phase; a higher volume of CE presented by NCCT indicates more serious blood–brain barrier damage, which was associated with a higher risk of ICH (26). Second, the toxicity of contrast agents could damage blood–brain barrier and cause contrast-induced encephalopathy; more extravasation of CE in the brain means more toxicity, having a higher risk of causing contrast encephalopathy (27). Third, the disruption of blood–brain barrier increases neuroinflammation and causes a deteriorating microenvironment, which accelerates neuronal death and white matter injury, creating a detrimental feedback loop (28). Fourth, a larger volume of CE may be involved in the more serious and extensive disruption of blood–brain barrier and cerebral tissue, which results in poor functional outcomes and increased risk of hemorrhagic transformation (29, 30).

Several limitations should be noticed when interpreting our findings. First, this was a retrospective study with a relatively small sample size in a single center. Some biases in patient selection and treatment procedures could not be fully controlled. Second, a recent study showed that dual-energy CT allows an accurate distinction between CE and ICH (31). However, our current findings may help to identify the risk of ICH after EVT by easily available NCCT, even if the ICH occurred after receiving the early NCCT. Early identification of delayed postoperative ICH rather than immediate postoperative ICH may yield important information on the NCCT. This may be our focus in the future. Third, the volume of CE may be affected by the technique of surgeons and the amount of contrast media used during EVT. Although we controlled several key confounding variables, the information on contrast dose was not collected, which may lead to heterogeneity in our results. Finally, spatial distribution differences were not incorporated into the statistical models. The present study specifically evaluates whether CE volume constitutes an independent risk factor for these outcomes, with analytical models deliberately omitting spatial parameters to focus on volumetric characteristics. Subsequent studies with a larger cohort will incorporate both volume and spatial distribution analyses of CE to better characterize their synergistic impacts on intracranial hemorrhage and long-term functional prognosis.

In conclusion, a larger volume of CE on NCCT obtained immediately after EVT in AIS-LVO patients may be independently associated with sICH and functional independence. The predictive performance of CE volume plus traditional variables for sICH and functional outcomes was significantly higher than a conventional model.

## Data Availability

The raw data supporting the conclusions of this article will be made available by the authors, without undue reservation.
