# Supplementary material for: Quantitative contrast enhancement volume on immediate post-thrombectomy CT predicts symptomatic intracranial hemorrhage and functional outcomes in acute large vessel occlusion stroke
Source: Front Neurol. 2025 May 29;16:1579659. doi: 10.3389/fneur.2025.1579659 (PMC12158709; doi:10.3389/fneur.2025.1579659)
Supplement: Supplementary file 1 [file Supplementary_file_1.docx]

**Quantitative Contrast Enhancement Volume on Immediate Post-Thrombectomy CT Predicts Symptomatic Intracranial Hemorrhage and Functional Outcomes in Acute Large Vessel Occlusion Stroke**

Figure S1. Calibration curve of multivariable models for determining symptomatic intracranial hemorrhage (A) and functional independence (B). The calibration curve shows how well the predicted probabilities agreed with the observed probabilities. The diagonal line represents a perfect prediction by an ideal model. The orange or pink line represents the predictive performance of the multivariable models. The orange or pink line has a closer fit to the diagonal line, which indicates a better prediction. The green or blue dotted line represents actual occurrence rate of symptomatic intracranial hemorrhage (sICH) and functional independence.


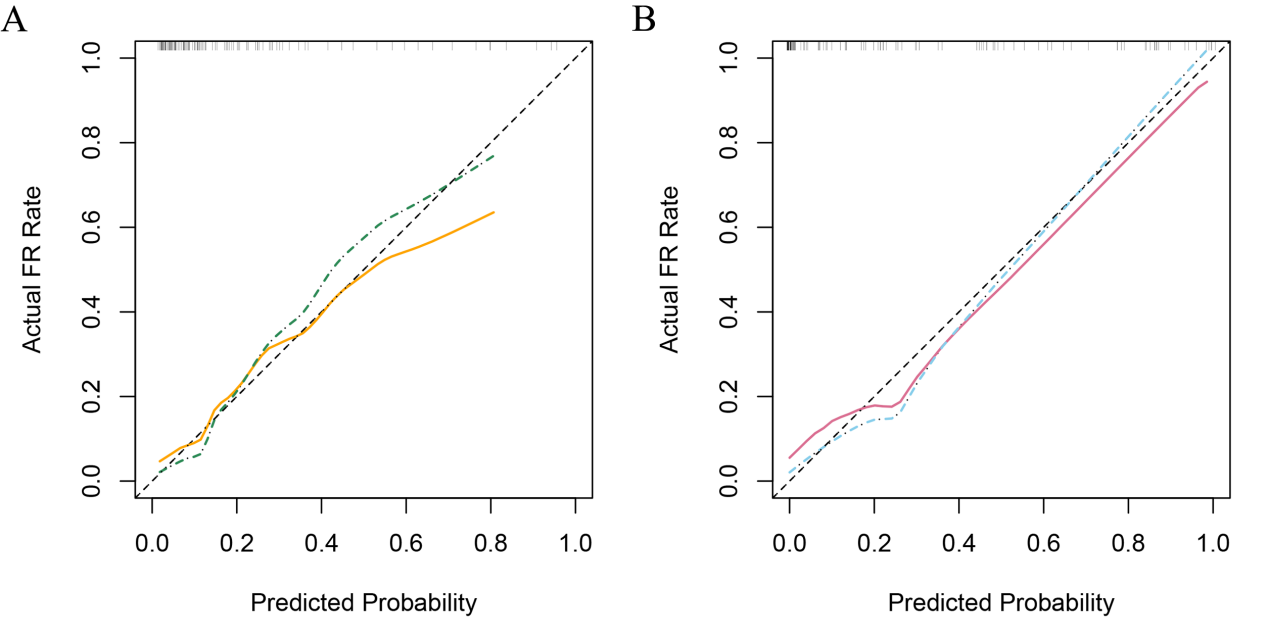


Figure S2. Decision curve analyses depicting the clinical benefit of predictive models for symptomatic intracranial hemorrhage (A) and functional independence (B) in full sample. The gray line represents the assumption that all patients have symptomatic intracranial hemorrhage (sICH) and functional independence, meaning 'treating all patients'. The black line (parallel to the x axis) indicates that no patients have above outcomes, meaning 'treating no patients'. The orange and pink line respectively represents prediction of the *sICH model 1 and Functional independence model 1* to guide treatment, and the green and blue line represent the traditional models. Both *sICH model 1 and Functional independence model 1* provide more accuracy than the traditional models, Treat all, and Treat none.


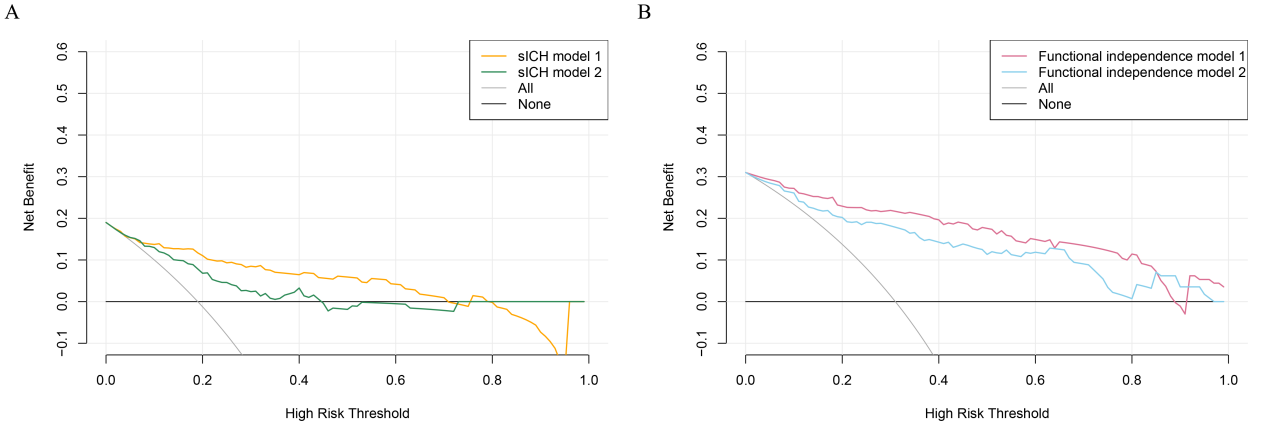


**Table S1**. Baseline characteristics according to groups of 90-day mRS 0-2 and 3-6

| **Characteristics** | **All**  **(n=111)** | **mRS 0-2**  **(n=35)** | **mRS 3-6**  **(n=76)** | **P value** |
| --- | --- | --- | --- | --- |
| Age, median (IQR) | 67（60-77） | 62（51-70） | 73（64-79） | <0.001 |
| Sex, n (%) | 57（51.4） | 20（57.1） | 37（48.7） | 0.407 |
| Medical history, n (%) |  |  |  |  |
| Smoking | 43（38.7） | 16（45.7） | 27（35.5） | 0.306 |
| Drinking | 35（31.5） | 12（34.3） | 23（30.3） | 0.672 |
| Hypertension | 55（49.5） | 15（42.9） | 40（52.6） | 0.339 |
| Diabetes mellitus | 19（17.1） | 7（20.0） | 12（15.8） | 0.584 |
| Atrial fibrillation | 49（44.1） | 12（34.3） | 37（48.7） | 0.156 |
| Clinical characteristics |  |  |  |  |
| SBP admission, mmHg; mean (SD) | 140（129-159） | 139（127-151） | 146（130-163） | 0.248 |
| DBP admission, mmHg; mean (SD) | 80（74-90） | 80（75-88） | 82（74-93） | 0.407 |
| blood glucose, median (IQR) | 7.2（6.2-8.8） | 7.5（6.3-9.5） | 6.9（6.1-8.4） | 0.369 |
| APTT, median (IQR) | 34.7（31.8-37.9） | 34.4（32.4-37.0） | 34.7（31.3-38.2） | 0.870 |
| PT, median (IQR) | 13.4（12.9-15.2） | 13.4（12.8-14.8） | 13.5（12.9-15.7） | 0.685 |
| INR, median (IQR) | 0.99（0.95-15.2） | 0.99（0.95-1.10） | 0.99（0.94-1.10） | 0.820 |
| Pre-stroke mRS, median (IQR) | 0（0-0） | 0（0-0） | 0（0-0） | 0.629 |
| TOAST, n (%)  Large atherosclerotic stroke  Cardiogenic stroke  Other | 52（46.8）  54（48.6）  5（4.5） | 19（54.3）  15（42.9）  1（2.9） | 33（43.4）  39（51.30）  4（5.3） | 0.531 |
| Baseline NIHSS score, median (IQR) | 15（10-20） | 11（7-15） | 16（11-21） | <0.001 |
| Baseline ASPECTS, median (IQR) | 6（5-7） | 7（6-8） | 6（5-7） | <0.001 |
| ASITN/SIR score, median (IQR) | 2（0-2） | 2（1-3） | 1（0-2） | 0.003 |
| eTICI 2b-3, n (%) | 97（87.4） | 64（84.2） | 33（94.3） | 0.239 |
| OTP, min; median (IQR) | 421（260-749） | 425（321-845） | 420（237-729） | 0.316 |
| OTR, min; median (IQR) | 525（373-844） | 529（381-794） | 540（375-919） | 0.621 |
| Intravenous thrombolysis, n (%) | 50（45.0） | 37（48.7） | 13（37.1） | 0.256 |
| Occlusion site, n (%)  ICA  MCA-1  MCA-2 | 33（29.7）  67（60.4）  11（9.9） | 7（20.0）  25（71.4）  3（8.6） | 26（34.2）  42（55.3）  8（10.5） | 0.252 |
| CE volume, median (IQR) | 10.6（5.2-42.1） | 5.4（2.9-9.6） | 20.9（8.2-68.6） | <0.001 |

mRS, modified Rankin Scale; SBP, [systolic](javascript:;) [blood pressure](javascript:;); DBP, diastolic [blood pressure](javascript:;); APTT, activated partial thromboplastin time; PT, prothrombin time; INR, international normalized ratio; mRS, modified Rankin Scale; TOAST, Trial of ORG 10172 in acute stroke treatment; NIHSS, National Institutes of Health Stroke Scale; ASPECTS, Alberta Stroke Program Early CT Score; ASITN/SIR, American Society of Interventional and Therapeutic Neuroradiology/Society of Interventional Radiology; eTICI, extended thrombolysis in cerebral infarction; OTP, the time from stroke onset to groin puncture; OTR, the time from stroke onset to revascularization; ICA, internal carotid artery; MCA, middle cerebral artery; SD, standard deviation; IQR, interquartile range; CE, contrast enhancement

**Table S2**. Univariable and multivariable logistic regression analysis of predictors for sICH

|  | **OR** | **95% CI** | **P value** | **Adjust OR** | **95% CI** | **P value** |
| --- | --- | --- | --- | --- | --- | --- |
| Age | 1.00 | 0.96-1.04 | 0.962 | 0.98 | 0.94-1.02 | 0.313 |
| Pre-stroke mRS | 1.01 | 0.47-2.17 | 0.989 | 0.65 | 0.28-1.53 | 0.325 |
| Baseline NIHSS score | 1.08 | 1.02-1.15 | 0.016 | 1.02 | 0.95-1.10 | 0.547 |
| Baseline ASPECTS | 0.74 | 0.56-0.98 | 0.038 | 0.95 | 0.68-1.34 | 0.783 |
| ASITN/SIR score | 0.47 | 0.29-0.77 | 0.002 | 0.52 | 0.29-0.93 | 0.028 |
| eTICI 2b-3 | 0.89 | 0.23-3.52 | 0.872 | 0.85 | 0.17-4.06 | 0.837 |
| Occlusion site, n (%)  ICA  MCA-1  MCA-2 | 0.35  0.20 | Reference  0.13-0.94  0.02-1.77 | 0.038  0.148 | 0.83  0.58 | Reference  0.25-2.77  0.06-6.19 | 0.759  0.655 |
| CE_-_/CE_+_ | 6.32 | 1.98-20.23 | 0.002 | 5.24 | 1.45-18.99 | 0.012 |

sICH, symptomatic intracranial hemorrhage; mRS, modified Rankin Scale; NIHSS, National Institutes of Health Stroke Scale; ASPECTS, Alberta Stroke Program Early CT Score; ASITN/SIR, American Society of Interventional and Therapeutic Neuroradiology/Society of Interventional Radiology; eTICI, extended thrombolysis in cerebral infarction; ICA, internal carotid artery; MCA, middle cerebral artery; CE, contrast enhancement CI confidence interval; OR, odds ratio

**Table S3**. Univariable and multivariable logistic regression analysis of predictors for functional independence

|  | **OR** | **95% CI** | **P value** | **Adjust OR** | **95% CI** | **P value** |
| --- | --- | --- | --- | --- | --- | --- |
| Age | 0.94 | 0.90-0.97 | <0.001 | 0.88 | 0.83-0.94 | <0.001 |
| Pre-stroke mRS | 0.85 | 0.41-1.77 | 0.661 | 2.32 | 0.86-6.24 | 0.095 |
| Baseline NIHSS score | 0.85 | 0.78-0.92 | <0.001 | 0.84 | 0.74-0.96 | 0.008 |
| Baseline ASPECTS | 1.65 | 1.22-2.24 | <0.001 | 1.95 | 1.18-3.22 | 0.009 |
| ASITN/SIR score | 1.75 | 1.22-2.52 | 0.003 | 0.94 | 0.54-1.63 | 0.813 |
| eTICI 2b-3 | 3.09 | 0.65-14.65 | 0.155 | 6.49 | 0.97-43.43 | 0.054 |
| Occlusion site, n (%)  ICA  MCA-1  MCA-2 | 2.21  1.39 | Reference  0.84-5.83  0.29-6.68 | 0.109  0.679 | 0.35  0.27 | Reference  0.08-1.55  0.03-2.40 | 0.165  0.238 |
| CE_-_/CE_+_ | 0.11 | 0.04-0.31 | <0.001 | 0.05 | 0.01-0.28 | <0.001 |

mRS, modified Rankin Scale; NIHSS, National Institutes of Health Stroke Scale; ASPECTS, Alberta Stroke Program Early CT Score; ASITN/SIR, American Society of Interventional and Therapeutic Neuroradiology/Society of Interventional Radiology; eTICI, extended thrombolysis in cerebral infarction; ICA, internal carotid artery; MCA, middle cerebral artery; CE, contrast enhancement CI confidence interval; OR, odds ratio

**Table S4**. VIF and tolerance values of independent variables for primary outcomes

| **Variables** | **Tolerance** | **VIF** |
| --- | --- | --- |
| Age | 0.946 | 1.057 |
| Pre-stroke mRS | 0.968 | 1.034 |
| Baseline NIHSS score | 0.723 | 1.383 |
| Baseline ASPECTS | 0.824 | 1.214 |
| ASITN/SIR score | 0.754 | 1.326 |
| eTICI 2b-3 | 0.959 | 1.043 |
| Occlusion site | 0.822 | 1.216 |
| CE volume | 0.785 | 1.273 |

VIF, variance inflation factor; mRS, modified Rankin Scale; NIHSS, National Institutes of Health Stroke Scale; ASPECTS, Alberta Stroke Program Early CT Score; ASITN/SIR, American Society of Interventional and Therapeutic Neuroradiology/Society of Interventional Radiology; eTICI, extended thrombolysis in cerebral infarction; CE, contrast enhancement

**Table S5**. Baseline characteristics according to Groups of CE_+_ and CE_-_ in consideration of antithrombotic drugs usage and mean SBP within 48h after EVT

| **Characteristics** | **All**  **(n=111)** | **CE_+_**  **(n=55)** | **CE_-_**  **(n=56)** | **P value** |
| --- | --- | --- | --- | --- |
| Antithrombotic drugs usage, n (%) | 39 (35.1) | 14 (25.5） | 25 (44.6) | 0.034 |
| Mean SBP within 48h after EVT, mmHg; mean (SD) | 126 (115-135) | 129 (118-137) | 123 (111-133) | 0.032 |
| Mean DBP within 48h after EVT, mmHg; mean (SD) | 73 (66-78) | 74 (68-79) | 72 (64-78) | 0.101 |

CE_+_, defined as CE volume ≥ 10.6 ml; CE_-_, defined as CE volume < 10.6 ml; SBP, [systolic](javascript:;) [blood pressure](javascript:;); DBP, diastolic [blood pressure](javascript:;); SD, standard deviation; IQR, interquartile range; CE, contrast enhancement

**Table S6**. Multivariable logistic regression analysis for sICH and functional independence in consideration of antithrombotic drugs usage and mean SBP within 48h after EVT

|  | **sICH** | | | **Functional independence** | | |
| --- | --- | --- | --- | --- | --- | --- |
|  | **Adjust OR** | **95% CI** | **P value** | **Adjust OR** | **95% CI** | **P value** |
| Antithrombotic drugs usage | 0.18 | 0.03-1.02 | 0.041 | 1.70 | 0.47-6.09 | 0.418 |
| Mean SBP within 48h after EVT | 0.98 | 0.95-1.02 | 0.409 | 0.97 | 0.93-1.01 | 0.112 |
| Age | 0.98 | 0.95-1.03 | 0.474 | 0.88 | 0.82-0.94 | <0.001 |
| Pre-stroke mRS | 0.65 | 0.28-1.51 | 0.474 | 2.53 | 0.90-7.08 | 0.078 |
| Baseline NIHSS score | 1.02 | 0.94-1.11 | 0.578 | 0.84 | 0.73-0.96 | 0.011 |
| Baseline ASPECTS | 0.96 | 0.67-1.38 | 0.832 | 2.11 | 1.25-3.58 | 0.005 |
| ASITN/SIR score | 0.55 | 0.29-1.05 | 0.069 | 1.01 | 0.56-1.82 | 0.975 |
| eTICI 2b-3 | 0.44 | 0.08-2.60 | 0.367 | 5.08 | 0.69-37.32 | 0.110 |
| Occlusion site, n (%)  ICA  MCA-1  MCA-2 | 0.87  0.61 | Reference  0.24-3.11  0.05-6.80 | 0.829  0.684 | 0.32  0.27 | Reference  0.07-1.48  0.03-2.47 | 0.146  0.245 |
| CE_-_/CE_+_ | 4.56 | 1.17-17.74 | 0.029 | 0.07 | 0.01-0.34 | 0.001 |

sICH, symptomatic intracranial hemorrhage; SBP, [systolic](javascript:;) [blood pressure](javascript:;); mRS, modified Rankin Scale; NIHSS, National Institutes of Health Stroke Scale; ASPECTS, Alberta Stroke Program Early CT Score; ASITN/SIR, American Society of Interventional and Therapeutic Neuroradiology/Society of Interventional Radiology; eTICI, extended thrombolysis in cerebral infarction; ICA, internal carotid artery; MCA, middle cerebral artery; CE, contrast enhancement CI confidence interval; OR, odds ratio
